# Fats, Friends or Foes: Investigating the Role of Short- and Medium-Chain Fatty Acids in Alzheimer’s Disease

**DOI:** 10.3390/biomedicines10112778

**Published:** 2022-11-01

**Authors:** Aishat O. Ameen, Kristine Freude, Blanca I. Aldana

**Affiliations:** 1 Department of Drug Design and Pharmacology, Faculty of Health and Medical Sciences, University of Copenhagen, 2100 Copenhagen, Denmark; 2 Department of Veterinary and Animal Sciences, Faculty of Health and Medical Sciences, University of Copenhagen, 1870 Frederiksberg, Denmark

**Keywords:** neurodegeneration, energy metabolism, hiPSC, octanoic acid, decanoic acid, butyrate

## Abstract

Characterising Alzheimer’s disease (AD) as a metabolic disorder of the brain is gaining acceptance based on the pathophysiological commonalities between AD and major metabolic disorders. Therefore, metabolic interventions have been explored as a strategy for brain energetic rescue. Amongst these, medium-chain fatty acid (MCFA) supplementations have been reported to rescue the energetic failure in brain cells as well as the cognitive decline in patients. Short-chain fatty acids (SCFA) have also been implicated in AD pathology. Due to the increasing therapeutic interest in metabolic interventions and brain energetic rescue in neurodegenerative disorders, in this review, we first summarise the role of SCFAs and MCFAs in AD. We provide a comparison of the main findings regarding these lipid species in established AD animal models and recently developed human cell-based models of this devastating disorder.

## 1. Introduction

Alzheimer’s disease (AD) is a growing global health challenge. AD is strongly linked to unhealthy lifestyles typified by detrimental diets and sedentary behaviour [1]. Although the cognitive dysfunction characterising AD is correlated with the accumulation of amyloid-β peptide (Aβ) deposits as well as tau-derived pathology [2], the molecular mechanisms underlying AD development are still not fully understood. Mounting evidence indicates that neurodegenerative disorders, particularly AD, are associated with metabolic dysfunctions [3]. Importantly, impairments in cerebral glucose and lipid metabolism have been demonstrated in major neurodegenerative disorders [4]. However, whether disturbed brain lipid metabolism leads to the cognitive decline in AD remains to be determined.

The strong association between the risk of developing AD and genes involved in lipid processing, such as apolipoprotein E4 (APOε4) [5], suggests that altered lipid metabolism could be a critical contributing factor leading to disease onset and progression. Brain and blood lipid composition analyses have revealed changes in specific lipid types in AD. These findings combined with the impaired brain uptake and utilisation of glucose, the necessary energy substrate in the brain, have provided a rationale to investigate the therapeutic potential of fatty acid-based metabolic interventions to restore brain function. The brain lipid profile can be directly influenced by dietary administration of fatty acids, and the gut microbiota may also serve as a source of beneficial lipids, particularly short-chain fatty acids (SCFAs), that may have a role in improving cognition [6,7].

In this review, we discuss the role of specific lipids in the brain, namely short- and medium-chain fatty acids (MCFAs) and their important association with AD. In addition, we provide an overview of the experimental evidence of the involvement of these fatty acids in animal and cellular models of AD pathology and highlight potential therapeutic avenues.

## 2. Alzheimer´s Disease Pathology Hallmarks

Alzheimer’s disease (AD) is a neurodegenerative disorder clinically defined as cognitive decline, memory loss, disorientation, and behavioural changes. Although therapies have been developed to treat patients with AD, none of these are currently able to cure the disease, creating an urgent need for better AD pathology models and interventions. AD can be divided into two main types based on the appearance of symptoms: early-onset AD and late-onset AD, both of which can be classified as familial AD (FAD) type and sporadic AD type, for which the underlying pathological mechanisms are different [8]. FAD is linked to inherited genetic mutations and accounts for 1–5% [9] of all AD cases worldwide and is associated with mutations to genes encoding amyloid precursor proteins (APP) and presenilin 1/2 (PSEN1/ PSEN2) [10].

AD is associated with the accumulation of amyloid-β peptide (Aβ) deposits and plaques which are produced from the cleavage of the transmembrane protein, APP [11] via the enzymes α, β, and γ secretases [12]. One of the pathways to produce toxic Aβ is from the cleavage of APP by β- and then γ-secretase. PSEN1 and PSEN2 encode part of the γ-secretase complex [13] hence mutations can lead to the formation of Aβ fragments: Aβ43, Aβ42, Aβ40, Aβ38, and Aβ37, with Aβ42 being associated most with Aβ aggregation [14].

In sporadic AD, the specific pathological mechanisms underlying the production of Aβ oligomers and plaques is unknown; however, APOε4 remains the most significant genetic risk factor for late-onset AD [14]. APOε4 promotes Aβ seeding, oligomerisation, and brain deposition while also dysregulating the endosomal–lysosomal system, leading to further Aβ accumulation [15,16,17]. Additionally, APOε4 accelerates blood-brain barrier (BBB) disintegration and tau-associated neurodegeneration, hence APOε4 is one of the targets for AD animal and cellular models [18,19].

Neurofibrillary tangles (NFTs) are another kind of aggregated protein seen in the brains of AD patients [20]. These NFTs consist of neuronal hyper-phosphorylated filaments of the microtubule-associated protein tau MAPT [21]. Like Aβ, these proteins aggregate in the hippocampus and cortex which are affected by neuronal and synaptic loss in AD [19]. In addition to these neuropathological changes, there are also several pathophysiological disruptions that occur in AD including neuroinflammation, glial activation, and mitochondrial dysfunction. The deposition of plaques and NFTs initiate a neuroinflammatory response through the activation of microglia and astrocytes that react to disturbances such as aggregated proteins and promote the release of pro-inflammatory cytokines such as interleukin (IL)-1β, IL-6, and tumour necrosis factor alpha (TNF-α), in addition to reactive oxygen species (ROS) [22]. Changes to mitochondrial function and disruption to brain bioenergetics have been well documented as early events in AD progression [23]. Evidence suggests that accumulating Aβ binds to mitochondrial proteins, resulting in dysfunction and an increase in ROS formation [24]. However, emerging evidence shows that mitochondria might also affect AD through the metabolic reprogramming of glial cells [23]. 

Metabolic insufficiency manifests as a progressive decline in brain glucose uptake and metabolism, leading to energy deficits. These energy deficits are linked to further dysfunction of neurones and glia as well as the accumulation of neurotoxic proteins before the onset of cognitive decline and diagnosis [25,26]. As a result, brain metabolism recovery has been hypothesised as a route to slowing down and perhaps reversing the progression of AD [27,28,29]. In the brain, glucose is the primary fuel; however, brain cells can also consume alternative substrates for energy production, including amino acids, ketone bodies (KB), and fatty acids [30,31]. Increasing attention is being given to the effect of SCFAs and in particular MCFAs as brain energy substrates [32,33]. In addition to functioning as energy substrates, fatty acids and their metabolites also exhibit neuroprotective effects through multiple mechanisms, including anti-inflammation and alterations in gene expression [33].

### 2.1. Animal Models of AD Pathology

Transgenic mice are used extensively in research as models of specific pathological traits of human diseases [34]. Transgenic mice are genetically modified by the introduction of human genes into the mouse genome. Several transgenic mouse models of AD have been created by the introduction of human FAD mutations in the APP and genes PSEN1/2 genes of the mouse genome. This introduction gives rise to the development of cerebral amyloid beta (Aβ) pathology in mice [35,36]. Conversely, no definitive human mutation in tau has yet been linked to AD; however, efforts have been made to develop transgenic mice expressing human microtubule-associated protein tau (hMAPT) with frontotemporal dementia-causing mutations [37,38]. When crossed with FAD models, these models have been shown to result in the formation of plaques and then NFTs, similar to the disease progression observed in AD patients [39].

In addition to rodents, AD has also been modelled in a variety of animals such as, dogs, pigs, and primates. These larger animal models tend to have longer lifespans and the ability to develop Aβ plaques and NFTs over time [40,41]. For example, the common marmoset is a primate with a lifespan of 7–17 years capable of developing plaques, tangles, and cognitive decline at 8 years old [21,42]. The appearance of these proteins can also be accelerated through the introduction of FAD mutations however these newer models remain costly, less established, and can raise ethical concerns [34]. Consequently, greater emphasis remains on developing humanised mouse models [34].

Transgenic AD mouse models containing mutations to a single gene have been used in the research on SCFAs and MCFAs. The most common of these AD model mutations is **APP Swedish (KM670/671NL)** shown to increase Aβ levels by 6–8 fold more than WT APP [43]. Another example is the **Tg2576** mouse which overexpresses the human APP Swedish mutation, leading to synaptic loss at 4 months and the formation of Aβ plaques at 11–13 months of age [44]. Similarly, another single mutation to the APP gene V717I (**APP London** model) results in an increase of soluble Aβ in the brain at 3 months, later proceeding to amyloid plaques at 12–14 months [45]. The **J20** model also contains mutations to a single gene; however, it differs in its overexpression of human APP with two FAD mutations, Swedish and Indiana (Table 1). The combination of these two mutations results in a model that accumulates Aβ plaques and cognitive impairment sooner than Tg2576 and APP London [46]. An example of a tau model used to explore the effect of SCFAs in AD is **Tg4510**. These mice express human tau with a MAPT P301L mutation that is associated with familial frontotemporal dementia and results in NFTs by 4 months, while neuronal loss and brain atrophy appear at about 6 months [47]. Whereas this model produces high levels of NFTs, research has revealed that insertion of this gene disrupts several mouse genes, leading to exaggerated tau pathology which must be considered when using this model [48]. 

Mutations to both APP and PSEN1/ PSEN2 have been shown to result in mouse models that develop faster and more aggressive amyloidosis than the single APP mutation models as well as earlier cognitive decline and neuronal loss [36]. **PS/APP** is a double transgenic model with APP Swedish and PSEN1 M146L mutations. This combination causes accelerated plaque formation as well as greater amounts of Aβ accumulation and glial cell activation compared to Tg2576 (Table 1) [49]. The **APPswe/PS1dE9** model differs from PS/APP due to its chimeric mouse/human APP Swedish mutation and deletion of exon 9, while **APP/PS1** has a different PSEN1 mutation (L166P) [50,51].

The **5xFAD** model expresses five FAD mutations, (Table 1) which leads to aggressive accumulation of cerebral Aβ and a severe AD phenotype [52]. At 1.5 months of age, these mice have an intraneuronal accumulation of Aβ and after 2 months, Aβ plaques, astrogliosis, and synaptic loss are detected [52]. Across all these mouse models cognitive decline is observed before or at the onset of Aβ deposition. 

The **3xTgAD** model contains mutations in three different genes: APP Swedish, MAPT P301L, and PSEN1 M146V resulting in Aβ plaque and tangle pathology [53]. In this model intracellular Aβ arises at 3 months, plaques at 9 months, and progression to NFTs at 12 months [53]. The **SIRT3+/−APP PS1** model also consists of mutations to three different genes creating GABAergic neuron loss and neuronal network hyperexcitability which is also observed in AD patients [54]. 

Efforts have been made to develop AD mouse models which reduce artifacts introduced by the overexpression of APP. Over-expression of APP leads to an exaggerated phenotype that is not representative of AD pathology seen in humans [35]. Hence, a more recent AD mouse model, **APP NL-G-F KI**, has been developed [55]. This model expresses APP Swedish, APP Iberian, and APP Arctic at WT levels as seen in human patients by using the endogenous APP promoter for gene expression; meaning the AD effects seen are due to mutation and not overexpression from an artificial promoter [35]. This is a gene knock-in approach resulting in amyloidosis at 2 months, glial activation, and cognitive impairment [55].

### 2.2. Cellular Models of AD Pathology

Cell models have helped to shape what we know about normal and pathological conditions [36]. Although animal models have been integral to studying APP in AD, these have not translated into effective treatment against AD [56]. In the case of AD mouse models, most result in aggressive forms of AD based on the overexpression of FAD mutations (which only accounts for a small fraction of AD cases). However, these do not model the disease itself or its initiation in sporadic AD cases [56]. Human cell models could facilitate the research of AD pathogenesis in different cell subtypes and potentially offer a more personalised approach to AD treatment [57].

SCFAs and MCFAs have not been tested extensively in human cell models of AD but there has been preliminary research in neuroblastoma cells, astrocytes, and neurons derived from human induced pluripotent stem cells. The ability to test the effect of SCFAs and MCFAs on specific cell types, co-cultures, and potentially organoid models would give a more accurate representation of their mechanisms of action. Induced pluripotent stem cells possess the ability to become any type of cell in the human body which is of great interest in the study of brain cells (neurons and glial cells) and their role in the progression of AD. On the other hand, the question remains as to whether these models can accurately simulate the phenotype of ageing associated with AD [58].

## 3. SCFAs and Their Metabolites

**Figure 1:**
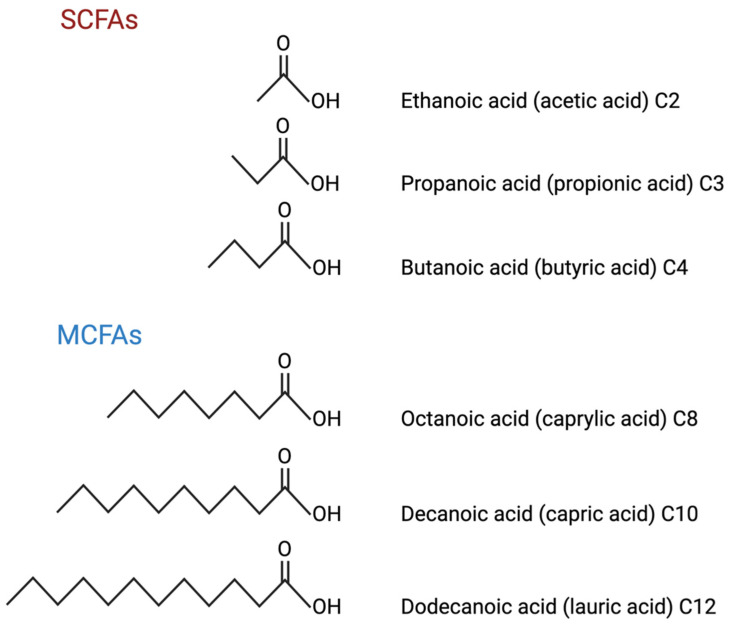
Examples of common SCFAs and MCFAs chemical structures and nomenclature. Figure created with BioRender.

SCFAs consist of 1–6 carbon atoms (Figure 1) and are formed from the fermentation of dietary fibre and saccharides in the gut by bacteria [32]. Propionate, butyrate, and succinate (a precursor of propionate and a crucial intermediate of the TCA cycle) are SCFAs generated by the gut microbiome and they affect the regulation of peripheral glucose metabolism, adipose deposition, and body weight [3]. Acetate which contains 2 carbons, is formed by reductive methylation of CO_2,_ while propionate (3 carbons) is produced through two routes. The first route is through the dicarboxylic pathway which results in propionate and acetate, the other is the acrylate pathway which converts lactoyl-CoA to propionyl-CoA and later propionate [32]. Butyrate (4 carbons) is formed from the condensation of two acetyl-CoA molecules, which are then converted to butyryl-CoA and subsequently butyrate [32,59]. In particular, the SCFA butyrate is absorbed by intestinal cells through monocarboxylate transporter 1 and sodium-coupled monocarboxylate transporter 1) to be utilised as a major energy source for these cells [60]. The remaining SCFAs are transported to the liver to be used as substrates in mitochondrial β-oxidation and the tricarboxylate cycle [59].

### Roles of SCFAs in AD

The ‘brain-gut-microbiome axis’ refers to a complex network of bidirectional communication between gut bacteria and the brain, and is crucial to maintain homeostasis of the gastrointestinal, central nervous, and microbial systems [61]. Recently, disruptions to this axis have been associated with a range of neurodegenerative disorders including Parkinson’s Disease (PD) and AD [6,62]. Human studies have shown that both AD and PD can be linked to changes in the levels of specific SCFAs and the bacteria that produce them [61,63]. AD mouse models have also revealed that levels of propionate and butyrate appear to be lower compared to wild-type mice [64].

Changes in histone acetylation and consequently gene expression have been linked to AD [6]. Butyrate can act as a histone deacetylase inhibitor and has been tested preclinically as a possible treatment for neurodegenerative diseases with abnormal histone acetylation [33].

SCFAs have been shown to affect neuroinflammation by inhibiting the expression of pro-inflammatory cytokines [65] while on the other hand promoting microglia maturation and function which could be beneficial in AD [66,67].

Like MCFAs, SCFAs can act as an energy substrate in the brain as shown in mouse models [68,69]. SCFAs might also be able to upregulate mitochondrial function in the brain; however, this has only been demonstrated in models for other neurological disorders; therefore, future research is needed to confirm this in AD [6].

## 4. MCFAs and Their Metabolites

MCFAs are monocarboxylic acids containing 6–12 carbon atoms (Figure 1) and are primarily derived from medium-chain triglycerides (MCT) [32,70]. MCTs obtained from the diet are broken down to MCFAs in the gastrointestinal tract by lipases and the MCFAs are absorbed through the gut wall to be circulated in the blood and transported to the liver [71]. In the liver, MCFA can undergo β-oxidation and subsequent ketogenesis resulting in the major ketone bodies (KB), β-hydroxybutyrate (BHB), acetoacetate (AcAc), and acetone (Ac) [25,71]. Consequently, KBs travel through the blood and can cross the BBB via monocarboxylate transporter 1, to act as an energy source in the brain [72].

Conversely, MCFAs are also transported directly to the brain and cross the BBB via diffusion and fatty acid transport proteins (FATPs) [73,74,75]. In astrocytes, MCFAs can undergo β-oxidization and subsequent ketogenesis in mitochondria [76,77,78]. The KBs produced by astrocytes can be transported to neurons for oxidative metabolism and studies suggest that astrocytes may be the main compartment for metabolism of the MCFAs, octanoic acid (C8), and decanoic acid (C10) [79,80].

### MCT-Based Metabolic Interventions for Energy Rescue in AD

Under certain circumstances, such as fasting or hypoglycaemia, FA or KB can be used by the brain for energy production [81,82]. A strategy to increase FA or KB levels in the brain is through dietary sources. The classic ketogenic diet, initially developed by Wilder in 1921, consists of 60–80% fat from long-chain fatty acids (LCFA), with 16–20 carbon atoms [83,84]. The low carbohydrate content of this diet makes it restrictive and difficult to maintain; hence, the development of an MCT-based diet in the 1950s [85]. In this diet, C8 and C10 are used instead of LCFAs. These fatty acids are more rapidly metabolised and generate larger ketone amounts compared to LCFAs [86,87].

MCT supplementation has been shown to be effective in treating drug-resistant epilepsy [88,89,90] and improving mild-to-moderate cognitive dysfunction in AD patients [3,91,92]. In humans, MCT diet supplementation caused a considerable increase in blood ketone levels and brain ketone consumption [93]. This increase is also demonstrated in animal and cell models [41,76]. C8 and C10 have a particularly positive effect on cognition [94,95]. A recent study using metabolic mapping, showed that C10 is preferred over C8 as a metabolic substrate in the cerebral cortex of NMRI mice, further supporting the idea of C10 supplementation as a metabolic intervention [96].

One study observed that some MCFAs may benefit brain health by modulating astrocyte metabolism, leading to the activation of shuttle systems in the form of lactate and ketone bodies that power neighbouring neurons. [97]. However, the significance of this shuttle as an energy source has been disputed by different studies showing that the energy derived from lactate is overestimated in most cases [98].

MCFAs could play a role in activating the free fatty acid sensing G-protein coupled receptors such as free fatty acid receptor 1 and 4 (FFAR1 and FFAR4) in the brain [99]. These receptors are involved in regulating energy metabolism and inflammation in different tissues [100]. For example, activation of FFAR1 in mitochondria has been implicated in enhancing mitochondrial respiration [101]. Furthermore, in an AD mouse model, activation of FFAR1 resulted in improved cognitive performance and the expression of neurotrophic factors associated with neurogenesis [102]. Together, these findings suggest MCFAs have roles beyond being an energy substrate.

## 5. Effect of SCFAs, MCFAs, and Their Metabolites on AD Mouse Models

### 5.1. Aβ Accumulation

In the J20 and APP V717I AD mouse models, treatment with BHB injection and a ketogenic diet respectively were able to reduce Aβ levels. Daily subcutaneous injection with BHB in the J20 model for 2 months led to a reduction in hippocampal amyloid plaque staining compared to control mice treated with saline [24]. Ketones were shown to decrease the accumulation of Aβ42 and increase the ratio of soluble Aβ40: Aβ42, supporting the production of less toxic Aβ40 [24,103]. In the APP V717I model, a study showed that when fed a ketogenic diet, these mice had lower levels of both Aβ40 and Aβ42. However, the ratio of Aβ40: Aβ42 did not change significantly between the mice fed a ketogenic diet and a control standard diet, suggesting that the diet did not change cleavage sites on APP, but instead aided overall lowering of Aβ species [45].

SCFAs have also been shown to reduce Aβ levels. In a 5xFAD model, mice were treated with prebiotic mannan oligosaccharide (MOS) to increase SCFA production after which their Aβ levels were assessed using immunofluorescent staining [104]. This study demonstrated that the mice receiving MOS had reduced amyloid formation in the hippocampus and amygdala compared to the control mice that were given normal drinking water [104]. Additionally, mRNA expression of APP and β-secretase (Bace1) in the cortex and hippocampus was reduced after MOS treatment. Another study in 5xFAD mice also used immunofluorescent staining to show that IP injection of sodium butyrate led to a decrease in Aβ accumulation in the hippocampus [105]. In a Tg2576 model, mice were injected with phenylbutyrate (PBA), and immunohistochemistry revealed levels of intraneuronal Aβ in the hippocampus decreased [106]. This could be due to PBA acting as a chaperone enhancing endoplasmic reticulum-folding function which favours the activity of the α-secretase ADAM10, an enzyme that catalyses the non-amyloidogenic cleavage of APP, consequently preventing Aβ formation [106].

Interestingly a study on the APP/PS1 model testing the effect of SCFA via drinking water was shown to increase Aβ deposition and plaque formation [107]. In this study, drinking water containing sodium propionate, sodium butyrate, and sodium acetate was given to germ-free (GF) mice from 4 weeks and specific pathogen-free (SPF) mice from 8 weeks until 12 weeks old. Immunoblot analysis GF mice brain tissue showed increased Aβ in the group given SCFA supplementation compared to the control. SCFAs did not seem to increase protein levels of APP secretases suggesting that increases in Aβ might be due to changes in clearance.

### 5.2. Neuronal and Synaptic Loss/Function

A 5xFAD model given MCT triheptanoin supplementation was shown to lessen synaptic loss. Mouse brain slices were co-stained for synaptophysin (a presynaptic marker) and PSD95 (a postsynaptic marker) to give an indication of synaptic density in the hippocampal CA1 region and entorhinal cortex. Triheptanoin 5xFAD mice displayed conserved synaptic density as well as expression levels of synaptophysin when compared to 5xFAD on a control diet; however, Aβ deposition remained unchanged [108] SIRT3^+/−^APP PS1 mice on a ketogenic diet containing BHB showed increased SIRT 3 expression and reduced loss of GABAergic neurons shown through immunoblot analysis [54].

In a different study, synaptic function of a 5xFAD model given the SCFA sodium butyrate (SB) was tested electrophysiologically by testing the long-term potentiation and depotentiation. long-term potentiation and depotentiation.was comparable across WT controls and 5xFAD given SB, suggesting retained synaptic plasticity [105] Golgi–Cox staining also revealed that dendritic spine density increased in the 5xFAD mice given SB. Additionally, western blots and immunofluorescence showed an increase in the expression of synaptic proteins in the 5xFAD mice treated with SB compared to controls [105]. In another study, 5xFAD mice treated with prebiotic MOS revealed clear morphological improvements in the prefrontal cortex, hippocampus, and amygdala, compared to untreated mice [104]. MOS appeared to reverse the shrinking of neurons and prevent further histological damage [104]. An increase in serum SCFAs was also associated with increased neuronal activity in the APP NL-G-F KI model. APP NL-G-F KI mice were given a probiotic supplement called VSL#3, which boosted levels of acetate butyrate, lactate, propionate, and isobutyrate as well as increasing in brain lactate and acetate [68]. These mice appeared to display greater neuronal activity in the hippocampus, demonstrated through a rise in c-Fos staining which is a marker of neuronal activation [68].

### 5.3. Neuroinflammation and Glia Activation

The APPswe/PS1dE9 and 5xFAD models have both been used to show that MCFAs and SCFAs have a positive effect on both neuroinflammation and glia activation [109] [65,104,105]. In APPswe/PS1dE9 mice on a ketogenic diet supplemented with MCTs, astrogliosis and the expression of pro-inflammatory cytokine interferon-γ was reduced, shown through immunofluorescence; however, microglia activation was not reduced [109].

In contrast, the SCFA sodium acetate in APPswe/PS1dE9 appeared to lower microglia activation. Immunohistochemistry in hippocampal regions revealed that a marker of activated microglia, CD11b, was lower in mice treated with sodium acetate compared to untreated mice; suggesting a role in neuroinflammation reduction [65]. The 5xFAD mice treated with prebiotic MOS were also shown to attenuate microglia activation which was demonstrated through reduced levels of ionised calcium binding adaptor molecule 1 (Iba-1), a marker of activated microglia [104]. The morphology of microglia was transformed from amoeboid microglia found in AD mice to ramified-type microglia upon treatment with MOS. Finally, the overexpression of two pro-inflammatory cytokines, TNF-α and IL-6, was reduced in MOS-treated mice, suggesting the amelioration of neuroinflammation [104]. Similarly, treatment of 5xFAD mice directly with SB reduced levels of TNF-α, IL-6, interleukin-1 beta (IL-1β), and Iba-1 in the cortex and hippocampus, compared to untreated AD mice [105].

Conversely, SCFA-supplemented germ-free APP/PS1 mice appeared to have more amoeboid-type microglia which is an indicator of activation [107]. A group used in-situ hybridisation and immunofluorescence to identify microglia via Cx3cr1 expression clustering around Aβ plaques [107]. They found that microglia surrounding Aβ plaques were increased in GF mice given SCFA. They later looked at the effect of SCFAs on phagocytosis and found that specific pathogen-free (SPF) APP/PS1 mice also produced more microglia clustering around Aβ; however, these microglia contained less intracellular Aβ when compared to control-treated SPF mice, suggesting weakened phagocytosis. This difference in phagocytosis, however, was not detected when tested ex vivo [107].

### 5.4. Mitochondrial Function

Studies on the effect of MCFAs and ketones on mitochondrial function have been conducted in the 5xFAD, 3xTgAD, APPswe/PS1dE9, APP/PS1, and J20. In 5xFAD mice treated with triheptanoin, the effect of triheptanoin on oxidative phosphorylation complexes was measured by isolating brain mitochondria from 5xFAD and using immunoblotting to detect key subunits and their amounts; however, no difference was observed between triheptanoin and control mice [108]. Markers of oxidative stress such as 4-hydroxynonenal (HNE), were lowered while levels of reduced glutathione (GSH) were restored, demonstrating the antioxidant effect of triheptanoin [108]. Respiration was then measured as an indicator of brain mitochondrial function. Mitochondrial complexes I/III/IV and II/III/IV were restored in triheptanoin-treated mice, presenting the positive effect of this MCT on mitochondrial bioenergetics. Similarly, a J20 mouse model treated with BHB improved mitochondrial function by restoring complex I activity [24]. Protein oxidation was also found to be decreased in the mice treated with BHB, illustrating the ability of ketones to protect against protein damage [24]. The 3xTgAD mice fed an ester of β -hydroxybutyrate and 1, 3 butane diol (KE) had a reduced ratio of free NADP+: NADPH in the cortex meaning an increase in the availability of NADPH; however, this change was not seen in the hippocampus [110]. This increase in NADPH was also seen in APP/PS1 mice given BHB and pyruvate [29]. In 3xTgAD mice, KE had no effect on ATP hydrolysis in the cortex but in the hippocampus, more ATP hydrolysis and energy release took place compared to AD mice given a control diet, suggesting restored metabolic function [110]. Mice on the KE and control diet had comparable levels of HNE in the cortex but it was reduced in the hippocampus of KE-fed mice, suggesting that ketone metabolism was highly effective in reducing free radical damage in regions prone to reactive oxygen species (ROS) [110]. In APPswe/PS1dE9 mice on a ketogenic diet supplemented with MCT, the expression levels of genes associated with energy metabolism were analysed and an increase was seen in the levels of some of these genes compared to AD mice on the control diet [109].

A study on the effect of SCFAs in 5xFAD mice revealed that being treated with prebiotic MOS reduced levels of oxidative stress in the brain compared to untreated mice [104]. Markers of oxidative stress tested were malondialdehyde (MDA), oxidised glutathione: reduced glutathione (GSSG: GSH) and 8-hydroxy deoxyguanosine (8-OHdG), which were all found to be significantly reduced in the brains of mice treated with MOS [104].

### 5.5. Cognitive Function

Of the ketones tested on AD mouse models, BHB was found to improve cognitive function in the J20 model compared to untreated mice [24]. The Morris Water Maze (MWM), which is associated with hippocampal synaptic plasticity, was used to test spatial learning and memory function [24,111]. AD mice treated with ketones performed similarly to WT and better than untreated AD mice [24]. The novel object recognition test (NOR) was then used to measure the effect of BHB on nonspatial memory and to assess working memory related to the frontal cortex and medial temporal lobe. The AD mice treated with BHB spent more time with the novel object than the untreated AD mice, suggesting that BHB improves learning and recognition memory [24].

The MWM test was also used to test the action of SCFAs in 5xFAD and APPswe/PS1dE9 mice. In the 5xFAD model, MOS increased SCFA production and these mice performed better in the MWM compared to untreated AD mice, suggesting improved cognitive and spatial memory [104]. A Y-maze test was conducted to investigate the effects of the MOS on working memory. AD mice and results showed MOS had no significant effects on working memory [104]. These results demonstrate that MOS and perhaps SCFAs, improve cognitive and spatial memory loss, but not working memory loss in 5xFAD mice [104]. The SCFA, sodium acetate, also improved the performance of APPswe/PS1dE9 mice in the MWM compared to untreated mice, again suggesting that SCFAs improve cognitive function in AD mice [104]. A study tested the effects of the SCFA, sodium butyrate (SB), on associative memory and learning in APP/PS1 mice [112]. To do this, treated and untreated mice were exposed to molecular and behavioural analysis such as Pavlovian fear conditioning which showed that SB treatment improved associative learning [112]. The qPCR analysis showed a significant upregulation of 8 out of 10 genes investigated in the hippocampus indicating that SB regulates genes associated with memory consolidation through increased hippocampal histone acetylation [112].

**Table 1:** Comparison of current AD mouse models studying SCFAs and MCFAs.

Model	Mutations	Neuropathology	Type of FA	Treatment Regime	Observations	Ref.
5xFAD	APP: Swedish (K670N/M671L) + Florida (I716C) + London (V717I) PSEN1: M146L + L286V	Aβ deposition and plaques at 2 months of age; astrogliosis; neuronal and synaptic loss	MCFA	MCT triheptanoin at 3.5 months old for 8 months	Restored brain ATP; preserved mitochondrial function; protection against synaptic loss in hippocampus and entorhinal cortex; no changes in amyloid depositions	[52,108]
			SCFA	Prebiotic mannan oligosaccharide at 6 months old for 8 weeks to increase SCFA production	Improved cognitive function and spatial memory; balanced brain redox status and suppressed neuroinflammatory responses; reduced the reduced Aβ in cortex, hippocampus, and amygdala	[52,104]
			SCFA	Sodium butyrate (salt of SCFA) IP injection at 8 weeks old for 2 weeks	Reduced neuroinflammation; improved synaptic plasticity; reduced Aβ	[52,105]
3xTgAD	APP: Swedish (K670M/N671L) PSEN1: M146V, MAPT: P301L	Aβ deposition at 3 months; plaques at 9 months; NFTs at 12 months; astrogliosis	KB	Ester of β -hydroxybutyrate and 1,3 butane diol at 8 months old for 8 months	Increased BHB levels in hippocampus and cortex; more reduced mitochondrial redox potential; lower level of oxidised lipids/proteins in hippocampus	[53,110]
APPswe/PS1dE9	APP: Swedish (K670M/N671L), PSEN1: exon 9 deletion	Aβ deposition and gliosis at 6–9 months; neuronal and synaptic loss	MCFA	Ketogenic diet supplemented with MCT triheptanoin at 3 months old for 3 months	Reduced astroglia response in vicinity of Aβ plaques; reduced expression of the pro-inflammatory cytokines in astrocytes; transcriptional up-regulation of the ROS detoxifying mechanisms Sirt1 and Pparg; no changes in amyloid deposition	[50,109]
			SCFA	Sodium acetate (salt of SCFA) oral gavage once daily for 4 weeks	Decreased cognitive impairment; anti-neuroinflammatory effects	[50,65]
APP/PS1	APP: Swedish (K670M/N671L), PSEN1: L166P	Aβ deposition and astrogliosis at 1.5 months; synaptic loss	KB	BHB and pyruvate at age 12–13 weeks for 5 weeks	Increased brain NADPH; Reduced neuronal hyperexcitability	[29,51]
			SCFA	Sodium butyrate (salt of SCFA) IP injection at 15 months old for 6 weeks	Improved associative memory; increased hippocampal histone acetylation and expression of genes linked to associative learning; no changes in amyloid deposition	[51,112]
			SCFA	SCFA drinking water containing sodium propionate, sodium butyrate, and sodium acetate given to germ-free mice from 4 weeks and specific pathogen-free mice at 8 weeks until 12 weeks old.	Increased Aβ deposition and plaque formation; increased microglia activation	[51,107]
PS/APP	APP: Swedish (K670N/M671L), PSEN1: M146L	Aβ deposition at 3 months; Aβ plaques at 6 months; gliosis	MCFA	Ketogenic diet containing MCT oil at 5 months old for 3 months	Increased locomotor activity; improved motor function; cognition not improved; no changes in amyloid deposition	[49]
SIRT3^+/−^APP PS1	APP: Swedish (K670M/N671L) PSEN1: exon 9 deletion, SIRT3: Heterozygous Knockout	Aβ deposition; degeneration of GABAergic neurons; seizure-related death before 5 months	KB	Ketogenic diet containing BHB at 4 months old for 2 weeks	Increased SIRT 3 expression; reduced loss of GABAergic neurons; prevented seizure related death	[54]
J2	APP: Swedish (K670M/N671L) + Indiana (V717F)	Aβ deposition and plaques at 5–7 months; gliosis; neuronal and synaptic loss	KB	BHB daily injection at 4 months old for 2 months	Reduced intracellular Aβ levels; rescued mitochondrial complex I activity; reduced oxidative stress; improved synaptic plasticity and cognition	[24,46]
APP V717I	APP: London (V717I)	Increased levels of soluble Aβ at 3 months; plaques at 10 months	KD	Ketogenic diet at 3 months old for 43 days	Reduced Aβ levels; no change in behaviour or cognition	[45,113]
Tg2576	APP: Swedish (K670M/N671L)	Aβ plaques at 11–13 months; synaptic loss at 4 months	SCFA	Phenylbutyrate (phenolic SCFA) injection at 6, 12 and 16 months old for 5 weeks	Increased clearance of intraneuronal Aβ; restored dendritic spine density in hippocampus; reduced ER stress	[44,106]
APP NL-G-F KI	APP homozygous knock-in: Swedish (K670M/N671L) + Iberian (I716F) + Arctic (E693G)	Aβ plaques at 2 months; gliosis	SCFA	probiotic VSL#3 supplement at 6–8 months old for 8 weeks	Increase in serum SCFAs acetate, butyrate, lactate, Propionate, and isobutyrate; increase in brain lactate and acetate; increased hippocampal c-Fos staining linked to increased neuronal activity	[55,68]
Tg4510	MAPT: P301L	NFTs by 4 months; neuronal loss, and brain atrophy at 6 months	MCFA	Ketogenic diet containing MCT oil at 5 months old for 3 months	Increased locomotor activity; improved motor function; cognition not improved; no changes in amyloid deposition	[47,114]

APP; amyloid precursor protein; BHB—beta-hydroxybutyrate; ER—endoplasmic reticulum; FAD—familial Alzheimer’s disease; IP—intraperitoneal injection; KB—ketone body; KD—ketogenic diet; KO—knock out; KI—knock in; MAPT—microtubule-associated protein tau; NADPH—nicotinamide adenine dinucleotide phosphate; PSEN-1—Presenilin 1; ROS—reactive oxygen species; SC—subcutaneous injection; SIRT 3—sirtuin 3; Tg—transgenic.

## 6. Effect of SCFAs and MCFAs on Cellular Models of Neurodegeneration and AD

### 6.1. Human Neuroblastoma

The effects of MCFAs and SCFAs have been studied in the human neuroblastoma cell line, SH-SY5Y, and have been shown to reduce oxidative stress, inflammation, and protect against cell damage caused by Aβ (Table 2).

A study demonstrated that C10 had the ability to reduce H_2_O_2_ and therefore confer neuroprotection through the reduction of oxidative stress [115]. To do this, Human SH-SY5Y cells were treated with phosphatidylcholine (a membrane phospholipid) containing C10 for 18 h, after which the level of H_2_O_2_ in the medium was measured using an Amplex Red (10-acetyl-3,7-dihydroxyphenoxazine) and horseradish peroxidase (HRP) assay [115]. Compared to the control, C10 significantly reduced H_2_O_2_ concentration, which was an effect also observed in mouse neuroblastoma cells [115]. It is possible that C10 is able to reduce H_2_O_2_ release by enhancing catalase activity in SH-SY5Y cells as this enzyme’s activity increased in cells treated with C10 [115]. The group also measured the effect of C10 on intracellular ROS and found that ROS levels were reduced in treated SH-SY5Y cells, indicating a neuroprotective pathway through which oxidative damage of cellular components is prevented and H_2_O_2_-induced cell death is avoided [115]. As ketogenesis does not take place neuroblastoma cells, the direct effect of C10, and not its metabolite BHB, can be measured. BHB levels were measured before and after treatment with C10 and the levels remained unaffected, indicating that the effect on cells is due solely to direct action from C10 and not its metabolite [115]. Together this data suggests that C10 might be able to ameliorate the oxidative damage seen in AD.

Another study in SH-SY5Y cells showed MCFA C8 to have a higher rate of β oxidation than C10 [116]. Cells were incubated with either glucose, C8, or C10, and later the rate of oxidation was measured via CO_2_ release from pyruvate dehydrogenase activity and the tricarboxylic acid (TCA) cycle. C8 β-oxidation was significantly higher than that of C10, by approximately 80% suggesting that C8 may be preferentially oxidised in SH-SY5Y cells [116]. Co-incubation with both C8 and C10 also highlighted the preference for C8 β-oxidation [116]. To determine the mechanism through which the rate of β-oxidation differed between the MCFAs; the carnitine shuttle system was investigated. MCFAs are known to be able to enter the mitochondrial matrix without carnitine, so to test this, the enzyme carnitine palmitoyl transferase I (CPT-1) was inhibited with etomoxir [116,117]. Under these conditions, C10 β-oxidation was found to be reduced by 95% in the presence of etomoxir while C8 β-oxidation was only inhibited by 34%. The carnitine shuttle system is the rate-limiting step in β-oxidation which indicates that C10 might be reliant on this system hence the lower rate of β-oxidation [116]. However, another study using mouse brain slices revealed similar rates of metabolism for both C8 and C10, independent of carnitine palmitoyl transferase I [79]. This could be due to differences in preparations as brain slices are more complex than SH-SY5Y cells, comprising several types of cerebral cells, such as astrocytes which are main cells of MCFA metabolism in the brain [79]

The SCFA, sodium propionate (SP), was shown to reduce inflammation and protect against cell damage from Aβ in SH-SY5Y cells [118]. Aβ causes inflammation in SH-SY5Y cells mediated by the transcription factor nuclear factor kappa B (NF-κB). SP treatment was shown to significantly reduce NF-κB translocation compared to untreated cells [118]. The viability of the cells was then measured after treatment with SP then Aβ and it was determined that the cells treated with SP were more viable compared to the control [118]

### 6.2. Mouse Neuroblastoma

In neuro2a cells, the SCFA SB had the effect of reducing oxidative stress as well as the expression of APP [119] (Table 2). To investigate the protective effects of SB on Neuro2a cells, viability after Aβ incubation was measured and it was discovered that SB protected against cell damage and death [119]. Using a ROS assay kit, researchers found that SB inhibited the production of Aβ-induced ROS thus maintaining mitochondrial function in an AD cell culture environment [119]. Finally, the effect of SB on gene expression was assessed using real-time qPCR (RT-PCR) which revealed that, compared to the control, SB had the greatest inhibitory effect on APP [119]. It has been postulated that this could be through the action of a g-protein-coupled receptor called GPR109A for which SB is a ligand [119,120]. GPR109A was found to be highly expressed after SB exposure while inhibition of GPR109A led to the subsequent recovery of APP expression, indicating that GPR109A might be important in the treatment of AD [119].

### 6.3. hiPSC Astrocytes and Neurons

Studies conducted in hiPSC-derived astrocytes revealed that β-oxidation of C8 was more effective than C10 at producing extracellular ketone bodies, BHB, and AcAc [76]. hiPSC astrocytes were incubated with uniformly labelled [U-^13^C] C8 or [U-^13^C] C10 then the release of labelled ketone bodies in the medium was measured over time, using liquid chromatography-mass spectrometry (LC-MS) [76]. The rate and amount of ketone release was higher in astrocytes, metabolising C8 compared to C10 [76]. Analysis of metabolic flux suggested that C8 and C10 had different β-oxidation pathways and that this could contribute to different biological functions such as C10 being more anticonvulsant than C8 [78,116].

Similarly, another group testing MCFAs in hiPSC astrocytes and neurons demonstrated a preference for C8 by astrocytes for ketogenesis [97]. The addition of C8 significantly increased the concentration of extracellular BHB compared to control astrocytes; however, this was not the case for C10. By contrast, C10 was shown to drive glycolysis in astrocytes more than C8. Real-time (seahorse assay) analysis of ATP production showed that inhibition of mitochondrial respiration did not reduce ATP levels in astrocytes while blocking glycolysis did reduce ATP, indicating a greater dependence on glycolysis in astrocytes [97,121]. C8 and C10 did not affect the synthesis of ATP in mitochondria; however, C10 increased the rate of lactate formation (a product of glycolysis). The study also found a reduction in mitochondrial membrane potential as well as a NADH in response to MCFAs in astrocytes but not neurons, indicating a reduction in mitochondrial respiration [97]. The differences seen between C8 and C10 were unexpected as different in length by only 2 carbons and oxidation of C10 should quickly produce C8, again suggesting different metabolic pathways for C8 (as a substrate for ketogenesis) and C10 (promoting glycolysis) [76,97]. One possible explanation could be that astrocytes express acyl-CoA dehydrogenases with a preference for shorter chain C8 over C10, resulting in a greater rate of β-oxidation and ketogenesis in response to C8 but this was not established in the study [97].

To better model late onset Alzheimer’s disease (LOAD) a study took dermal fibroblasts and blood samples from LOAD patients and healthy controls without AD and reprogrammed them to iPSCs [122]. Astrocytes derived from these iPSCs displayed bioenergetic changes in mitochondrial respiration and glycolysis, reduced levels of NAD/NADH and disrupted glucose uptake [122]. Using Seahorse mitochondrial stress testing, the group showed that BHB did not significantly improve mitochondrial respiration in astrocytes [122]. Yet as these tests were not conducted under conditions of reduced glucose or fasting (as tested in mice) it could be stopping astrocytes from shifting to mitochondrial respiration over glycolysis [121,122].

**Table 2:** Comparison of current cellular models studying SCFAs and MCFAs.

Model	Type of FA	Treatment Regime	Observations	References
Human SH-SY5Y	MCFA	Decanoic acid	Reduced oxidative stress; decrease in H_2_O_2_ induced cell death independent of BHB levels	[115]
	MCFA	Octanoic acid and decanoic acid	Higher rate of β-oxidation for C8 compared to C10; greater dependence of C10 on CPT1	[116]
Human SH-SY5Y + Aβ	SCFA	Sodium propionate (salt of SCFA)	Reduced inflammation; protected against cell damage from Aβ	[118]
Neuro2a cells	SCFA	Sodium butyrate (salt of SCFA)	Reduced oxidative stress; reduced expression of APP	[119]
hiPSC astrocytes	MCFA	MCFAs octanoic acid and decanoic acid	Greater extracellular concentrations and faster secretion rates of BHB and AcAc with C8 than C10	[76]
	MCFA	MCFAs octanoic acid and decanoic acid	Reduction in mitochondrial electrical potential; reduction in levels of NADPH; C10 increased glycolysis; C8 increased rate of astrocyte ketogenesis	[97]
hiPSC neurons	MCFA	MCFAs octanoic acid and decanoic acid	No significant change in metabolic function	[97]
hiPSC astrocytes (from late-onset AD patients)	KB	BHB	No significant change in metabolic function	[122]

AcAc—acetoacetate; AD—Alzheimer’s disease; APP; amyloid precursor protein; BHB—beta-hydroxybutyrate; C8—octanoic acid; C10—decanoic acid; CPT1—carnitine palmitoyl transferase; hiPSC—human induced pluripotent stem cell; H_2_O_2_—hydrogen peroxide; NADPH—nicotinamide adenine dinucleotide phosphate; PSEN-1—Presenilin 1; SH-SY5Y—neuroblastoma cell-line.

## 7. Conclusions

Overall, some SCFAs and MCFAs have been shown to ameliorate some of the hallmarks of AD in mouse models. Ketones and SCFAs had the effect of reducing Aβ accumulation or increasing Aβ clearance, neuronal and synaptic loss was reduced while cognitive function was repaired in some mouse models. Ketone bodies and MCFAs restored the function of mitochondrial complexes whereas MCFAs and SCFAs dampened both neuroinflammation and glia activation. SCFAs, MCFAs, and ketone bodies all showed evidence of reducing ROS. The relationship between AD and SCFAs, however, appear to be more complex with a recent study reporting increased Aβ deposition, plaque formation and microglia activation in response to SCFAs. Less research has been carried out in cells modelling AD; hence, the data are limited but what has been demonstrated is that MCFAs have a greater influence on hiPSC derived astrocytes than neurons. Differences in FA chain length also seemed to affect different metabolic pathways. C8 and C10 in neuroblastoma cell-lines tended to reduce inflammation, oxidative stress and increase β-oxidation. The effects of SCFAs and MCFAs in mouse models and cell-based models differ considerably, which could be due to the loss of cell complexity and networks in cell models. However, it is this same simplicity that allows us to decipher the mechanisms through which MCFAs and SCFAs might be functioning. Future testing of SCFAs and MCFAs in more representative animal AD models such as humanised mouse models is needed to establish the mechanisms of FA neuroprotection. There is also a need for more cell models that recapitulate some of the hallmarks of sporadic AD at different progression points. Current research, nonetheless, points to the benefits of MCFAs and SCFAs in a multi-targeted approach against AD.

## Data Availability

Not applicable.
